# A Compact Multiband Shark-Fin Antenna for Integrated V2X Communication Systems

**DOI:** 10.3390/s26102962

**Published:** 2026-05-08

**Authors:** Xiao Ding, Wende Zha, Botao Feng, Yijia Ou, Chow-Yen-Desmond Sim

**Affiliations:** 1 Faculty of Fine Arts and Design, Guangdong Vocational Academy of Art, Foshan 528000, China; dingx@gdddc.edu.cn; 2 College of Electronics and Information Engineering, Shenzhen University, Shenzhen 518060, China; 2070436067@email.szu.edu.cn (W.Z.); 2500042026@mails.szu.edu.cn (Y.O.); 3 Department of Electrical Engineering, National Sun Yat-sen University, Kaohsiung 80424, Taiwan

**Keywords:** vehicular communication systems, V2X, multiband vehicular antenna, integrated antenna design, shark-fin antenna

## Abstract

A compact multiband shark-fin antenna is proposed for integrated vehicle-to-everything (V2X) platforms. The design incorporates five radiating elements within a compact $90\times 15\times 30\;{\mathrm{mm}}^{3}$ footprint, simultaneously supporting FM (88–108 MHz), TETRA (380–470 MHz), wideband cellular (0.68–6.05 GHz), and dual-band Wi-Fi services. Wideband cellular operation is realized using two mirrored planar inverted-F antennas (PIFAs), while a dual-band IFA provides Wi-Fi connectivity for in-vehicle and vehicle-to-infrastructure communications. The FM and TETRA elements employ compact meandered-line configurations to satisfy stringent rooftop space constraints. To improve multi-radio coexistence, the FM radiator is strategically placed between the two cellular elements, achieving inter-element isolation better than −15 dB across all operating bands. Experimental results demonstrate stable radiation performance, with realized gains ranging from 1.5 dBi to above 5 dBi and cross-polarization levels below −13 dB, in good agreement with simulations. With overall dimensions of $90\times 15\times 30\;{\mathrm{mm}}^{3}$, the proposed antenna is well suited for integrated V2X applications.

## 1. Introduction

**Figure 1:**
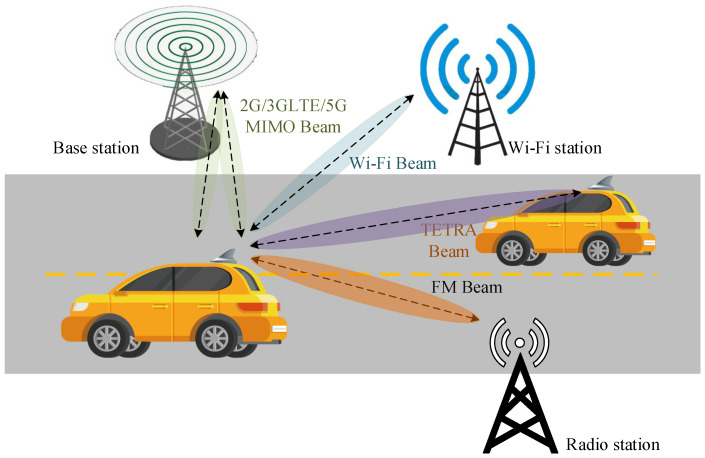
Integrated multi-service V2X communication scenario enabled by the proposed compact multiband shark-fin antenna.

With the rapid evolution of connected and autonomous vehicles, vehicular communication platforms are required to simultaneously support broadcast reception, professional mobile radio, wideband cellular connectivity, and short-range vehicle-to-everything (V2X) links, as shown in Figure 1 [1,2,3]. This convergence of heterogeneous standards significantly increases the demand for compact, multiband, and highly integrated rooftop antennas capable of maintaining stable radiation performance under electromagnetically complex vehicular environments. Among various form factors, the shark-fin configuration has become the de facto solution in modern vehicles due to its low profile, aerodynamic contour, mechanical robustness, and suitability for multi-antenna integration across sub-6 GHz and Wi-Fi bands [4,5,6]. However, strict constraints on volume and height, strong near-field coupling within confined enclosures, and the requirement for simultaneous low-frequency and wideband operation pose substantial challenges to antenna miniaturization and multi-radio coexistence [7,8].

From a problem-oriented perspective, existing compact vehicular antennas can be systematically categorized into three principal areas: radiator miniaturization under low-profile constraints, dense multi-antenna integration with isolation enhancement, and multiband/wideband realization across heterogeneous services.

*(1) Radiator miniaturization:* To accommodate limited rooftop height, PIFAs, IFAs, and meandered or folded monopoles have been extensively adopted [9,10]. For example, a compact PIFA reported in [9] achieved continuous 0.95–6 GHz coverage within a 54 × 38 × 25 mm^3^ volume by incorporating a wineglass-shaped slot and a switchable ground-ring slot, with peak gains of 7.08 dBi at 5.5 GHz and 6.65 dBi at 5.9 GHz (VSWR < 2). In [10], a shark-fin embedded monopole operating from 698 MHz to 6 GHz employed a 3.9 pF lumped capacitor to compensate for reduced radiator height, achieving efficiency above 63% below 1 GHz and above 68% in higher bands. Although these approaches effectively reduce physical size, aggressive miniaturization often increases the quality factor of electrically small radiators, thereby degrading impedance bandwidth and radiation efficiency, especially in sub-1 GHz regimes.

*(2) Dense multi-antenna integration and isolation control:* To satisfy Multiple-Input Multiple-Output (MIMO) and diversity requirements, multiple radiators are frequently arranged within a single shark-fin enclosure [11,12]. An orthogonally arranged three-dimensional quad-element configuration with a stub-loaded ground plane achieved isolation exceeding 20 dB, envelope correlation coefficient (ECC) below 0.12, and radiation efficiency above 90% across GSM, Bluetooth, and UWB bands [11]. A twelve-port integrated module reported in [12] demonstrated wideband operation; however, closely spaced monopoles exhibited $|S_{21}|$ approaching −6 dB under compact spacing. In the millimeter-wave regime, spatial orthogonality and defected ground structures (DGSs) have been exploited to enhance isolation. A four-port 24.6–31.8 GHz V2V MIMO antenna combining $90^{\circ }$-rotated elements with a concentric-ring DGS achieved isolation above 22.5 dB, peak gain of 8.1 dBi, and ECC below 0.007 within a 15 × 15 mm^2^ footprint [13]. Similarly, a half-mode substrate integrated waveguide (HMSIW)-based octagonal array at 28 GHz realized isolation exceeding 30 dB and gain up to 17.4 dBi without additional decoupling networks [14]. Intrinsic self-decoupling strategies have also been reported; by asymmetrically loading shorting pins on a TM^11^-mode patch at 5.9 GHz, isolation up to 78 dB was experimentally achieved over 5.82–5.98 GHz with 95% efficiency and ECC below 0.0003 at 0.14$\lambda_{0}$ spacing [15]. Despite these advances, translating such techniques to compact sub-6 GHz shark-fin platforms remains challenging due to volumetric limitations and broadband requirements.

*(3) Multiband and wideband coverage:* To simultaneously accommodate FM, TETRA, cellular, and Wi-Fi services, multiresonant excitation and bandwidth-enhancement techniques have been introduced. Slot loading, parasitic resonators, and metasurface superstrates enable excitation of multiple resonant modes within limited apertures [9,16]. A metasurface-assisted vehicular antenna reported in [16] achieved 1.7–5.7 GHz coverage (108% fractional bandwidth) with isolation above 27 dB and stabilized gain performance. Nevertheless, extending coverage toward FM (88–108 MHz) and TETRA (380–470 MHz) bands remains fundamentally constrained by the electrically small radiator condition and increased structural complexity when integrating widely separated frequency bands.

The above studies collectively indicate that achieving compactness, broadband sub-6 GHz coverage, acceptable low-frequency efficiency, and adequate inter-element isolation within a unified shark-fin enclosure remains a nontrivial system-level challenge.

To address the aforementioned limitations of compact size, wide low-frequency coverage, and high isolation, this work proposes a compact multiband shark-fin antenna system for integrated V2X-oriented communications. The main contributions are summarized as follows:

- A five-element integrated architecture is implemented within a 90 × 15 × 30 mm^3^ enclosure, simultaneously supporting FM (88–108 MHz), TETRA (380–470 MHz), wideband 2G–5G cellular services (0.68–6.05 GHz), and dual-band Wi-Fi (2.4/5.2 GHz), thereby enabling unified multi-standard rooftop deployment.
- A bandwidth-enhanced cellular subsystem employing mirrored wideband PIFAs is developed to provide quasi-omnidirectional radiation, cross-polarization levels below −13 dB, and realized gains up to 5 dBi across sub-6 GHz bands.
- Miniaturized low-frequency radiators are realized using a meandered TETRA monopole and a multilayer FR4-based FM element, while a dual-branch IFA without dielectric loading ensures Wi-Fi gains ranging from −0.4 to 4.8 dBi with limited fluctuation.
- A layout-driven isolation strategy is introduced by centrally embedding the dielectric FM radiator between the two cellular elements, maintaining inter-element isolation better than −15 dB across all operating bands without external decoupling networks.

The proposed design offers a compact, broadband, and integration-ready antenna solution for next-generation V2X vehicular platforms.

## 2. Antenna Geometry and Working Mechanism

### 2.1. Overall Configuration

**Figure 2:**
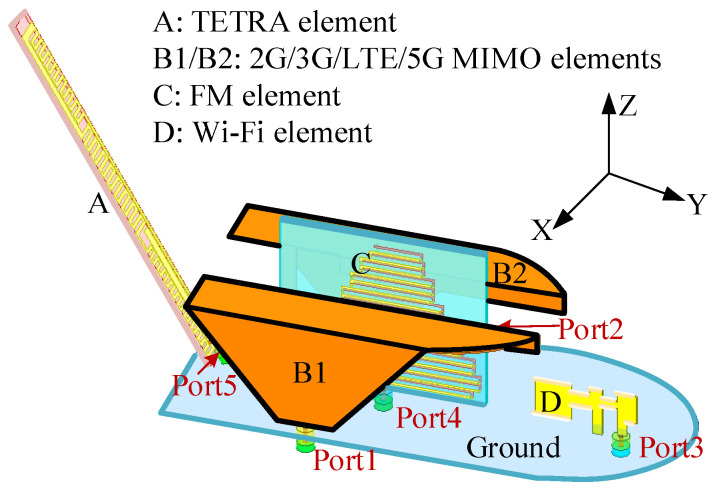
Overall configuration of the proposed shark-fin antenna.

The proposed antenna is integrated within a streamlined shark-fin enclosure to simultaneously support cellular, V2X, FM, TETRA, and Wi-Fi services under stringent volumetric constraints. As shown in Figure 2, five radiating elements are arranged to form four functional subsystems while preserving radiation stability and inter-element isolation. Element A is a rear-mounted TETRA monopole for V2V links. Elements B1 and B2 constitute a mirror-symmetric sub-6 GHz MIMO pair for 2G/3G/LTE/5G operation. Element C is a centrally positioned FM radiator, whose relatively larger height conforms to the enclosure profile and simultaneously acts as a spatial decoupling structure between the MIMO elements. Element D, located at the front, is a dual-band Wi-Fi antenna for in-vehicle and roadside connectivity. All elements share a common elliptical metallic ground plane to ensure electrical consistency. The TETRA and Wi-Fi antennas are fabricated on FR-4 substrates ($\varepsilon_{r}$ = 4.4 (the relative permittivity of the dielectric substrate), tanδ ≈ 0.02), whereas the cellular MIMO elements employ copper-sheet structures. This hybrid implementation balances fabrication cost, mechanical robustness, and radiation efficiency. It is worth noting that in practical deployments, the antenna’s sensitivity to vehicle roof variations depends on frequency. For high-frequency bands (sub-6 GHz and Wi-Fi), the localized elliptical ground plane ensures stable performance with minimal dependence on the vehicle roof geometry or material. For low-frequency bands (FM and TETRA), the extended vehicle roof acts as an effective ground plane, generally enhancing radiation efficiency and supporting resonance. In practical implementations, a conductive mounting surface or equivalent metallic layer is required to maintain stable low-frequency operation.

The structural dimensions of each proposed antenna were determined through a systematic two-step process. First, analytical methods were employed: the dimensions of the Wi-Fi and LTE elements were calculated using their corresponding equivalent circuit models, while the initial lengths of the meandered TETRA and FM radiators were estimated based on fundamental quarter-wavelength (λ/4) resonance theories. Subsequently, to account for the complex near-field mutual coupling between the closely packed metallic elements within the highly constrained shark-fin volume, a full-wave electromagnetic optimization was conducted. The Pattern Search algorithm within Ansys HFSS was utilized to meticulously fine-tune all physical parameters, thereby achieving the optimal balance between multiband impedance matching and high inter-element isolation.

### 2.2. Antenna Components

#### 2.2.1. 2G/3G/LTE/5G MIMO Antenna Element

**Figure 3:**
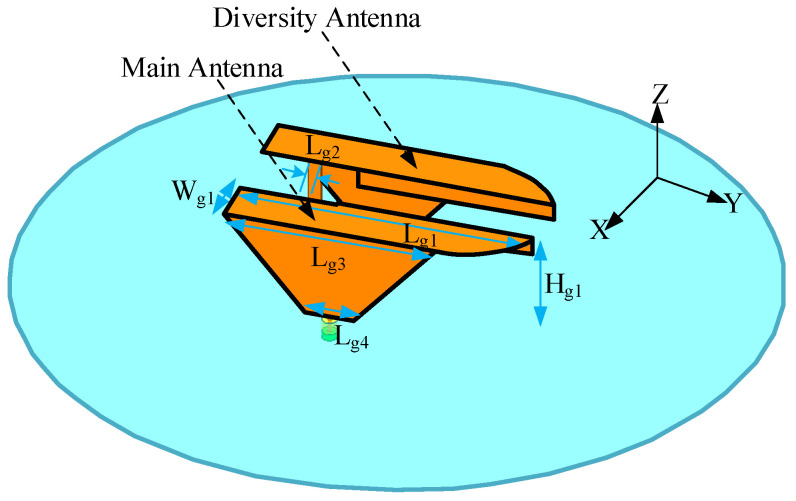
2G/3G/LTE/5G sub-6 GHz MIMO antenna element.

The sub-6 GHz MIMO subsystem (as depicted in Figure 3 and Table 1) consists of two mirrored PIFA-based radiators mounted above a metallic reflector. A 400-mm-radius ground plane is adopted to emulate the vehicular rooftop environment. Each element comprises a folded radiating strip, a shorting branch, and a feed. The radiator evolves from a monopole-like structure to a compact folded PIFA. A trapezoidal lower section introduces distributed capacitance, enhancing impedance bandwidth, while the upper folded branch reduces profile without sacrificing electrical length. Through geometric refinement, the element width is reduced from 25 mm to 15 mm and the height from 35 mm to 30 mm, while maintaining multiband coverage. The inter-element spacing increases from 10 mm to 22 mm, inherently improving isolation.

**Figure 4:**
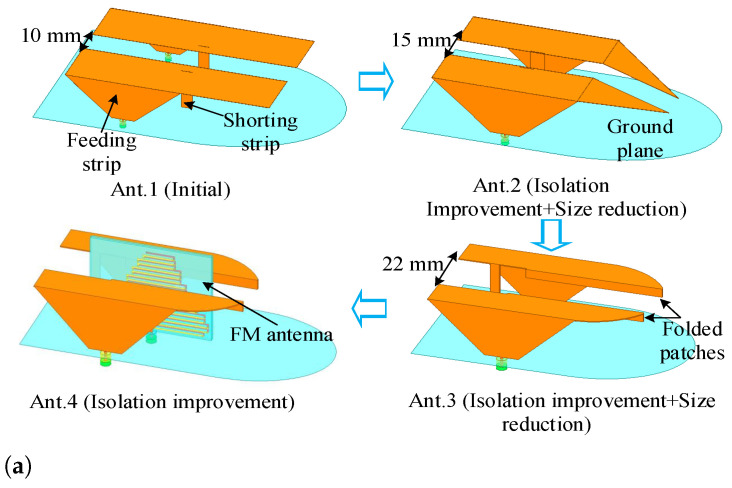

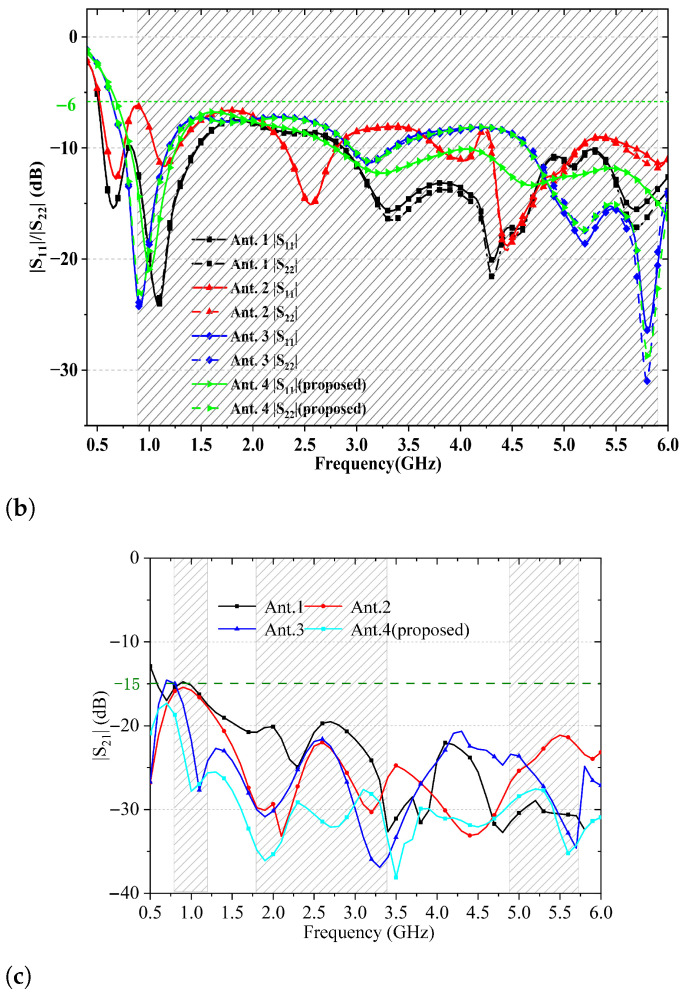
Design process and simulated S-parameters of the 2G/3G/LTE/5G MIMO antenna elements. (**a**) Design flow chart; (**b**) Reflection coefficients ($|S_{11}|$, $|S_{22}|$); (**c**) Isolation ($|S_{21}|$).

Figure 4 illustrates the evolution of the proposed sub-6 GHz 2G/3G/LTE/5G MIMO antenna. The initial design (Ant. 1) employs a broadband monopole-like branch covering 1.7–3.3 GHz and 5.1–5.5 GHz, while a tapered trapezoidal feed introduces the 0.6–1 GHz band. To satisfy the low-profile shark-fin constraint, the radiator is progressively folded and reshaped. In Ant. 3, the branch becomes a compact bent structure with an optimized shorting position, reducing the width from 25 mm to 15 mm and the height from 35 mm to 30 mm without altering the operating bands. The reduced footprint increases inter-element spacing from 10 mm to 22 mm, improving isolation to better than 16 dB. In the final configuration (Ant. 4), a vertical FM antenna is inserted between the two MIMO elements, serving both as a functional radiator and as a decoupling structure. As shown in Figure 4b, $|S_{11}|$ and $|S_{22}|$ remain below −6 dB across all target bands despite significant miniaturization. Figure 4c demonstrates progressive isolation enhancement, exceeding 20 dB across the operating range and below −30 dB over most frequencies. The results confirm that geometric refinement and integrated decoupling jointly suppress near-field coupling while preserving bandwidth.

**Figure 5:**
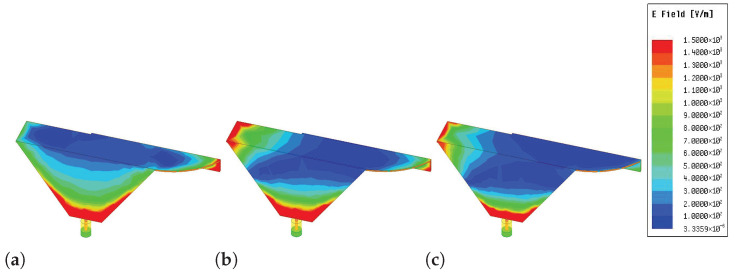
E-field of the 2G/3G/LTE/5G element. (**a**) 0.8 GHz; (**b**) 2.5 GHz; (**c**) 4.8 GHz.

Figure 5 presents the simulated surface electric-field distributions at 0.8, 2.5, and 4.8 GHz. At 0.8 GHz, strong fields extend over the entire structure, indicating a quarter-wavelength monopole-like mode governed by the overall current path. At 2.5 and 4.8 GHz, the fields become localized on specific radiating segments, revealing higher-order or local resonant modes. This frequency-dependent redistribution verifies the multi-resonant mechanism enabling compact multiband operation.

**Figure 6:**
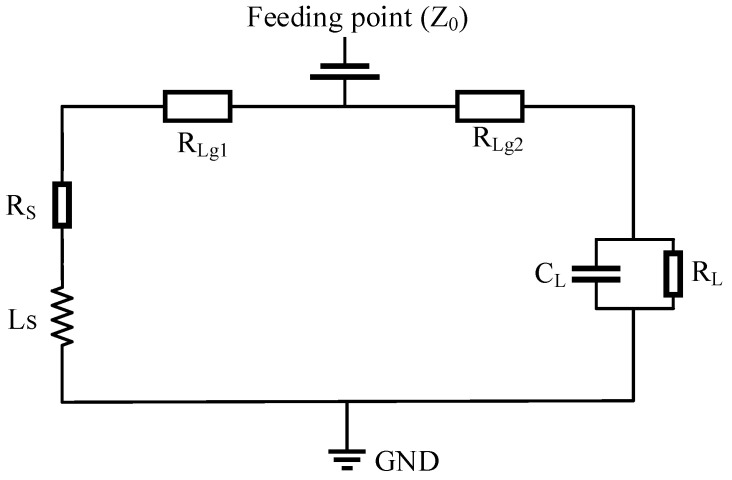
Equivalent-circuit of the proposed PIFA-shaped 2G/3G/LTE/5G antenna element.

The equivalent circuit of the PIFA-shaped element (Figure 6) further clarifies its operating principle. The fundamental resonance follows the quarter-wavelength approximation

(1) $$\begin{array}{c} f_{0}=\frac{c}{4\left(L_{g1}+H_{g1}+W_{g1}-L_{g2}\right)\sqrt{\varepsilon_{r}}}, \end{array}$$

where the effective current path determines the resonant frequency [17]. Here, $\varepsilon_{r}$ is defined as the relative permittivity of the dielectric substrate. The circuit can be interpreted as two parallel branches associated with the main radiating path and open-end reactive loading. The corresponding input admittance is

(2) $$\begin{array}{c} \frac{1}{Z_{0}}=\frac{1}{R_{Lg1}+R_{S}+j\omega L_{S}}+\frac{1}{R_{Lg2}+\frac{R_{L}}{1+j\omega C_{L}R_{L}}}. \end{array}$$

Here, $R_{s}$ and $L_{s}$ denote the parasitic resistance and distributed inductance of the ground plane, respectively. $R_{L}$ and $C_{L}$ represent the parasitic resistance and capacitance at the shorting end. Furthermore, $R_{Lg1}$ corresponds to the distributed resistance of the metallic trace in the shorting branch, while $R_{Lg2}$ denotes the distributed resistance of the metallic trace in the main radiating branch. This model explains the impedance-tuning flexibility and multiband behavior through controlled current-path modulation and reactive loading, providing a systematic basis for compact shark-fin antenna integration in V2X platforms.

**Figure 7:**
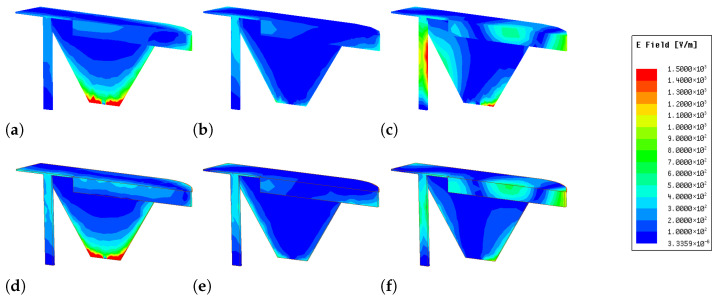
Simulated electric-field distributions of the coupled 2G/3G/LTE/5G antenna element. (**a**–**c**) Without the FM element at 0.8, 2.5, and 4.8 GHz, respectively; (**d**–**f**) with the FM element at the corresponding frequencies.

Specifically, Figure 7 illustrates the field distribution of one cellular antenna element (2G/3G/LTE/5G) when the adjacent element is excited, under two scenarios: with and without the FM element. As observed from the figure, at higher frequencies (e.g., 4.8 GHz), the presence of the FM element leads to a noticeable reduction in the coupled electric field. In contrast, at lower frequencies, the influence of the FM element on the field distribution is negligible. This comparison clearly demonstrates that the dielectric substrate and metallic traces of the FM element function as an effective spatial decoupling barrier. By perturbing the near-field coupling paths, the FM structure suppresses wave propagation between the two cellular antennas, particularly at higher frequencies. These results provide intuitive visual evidence that the proposed layout-driven strategy enhances inter-element isolation without the need for additional decoupling components.

#### 2.2.2. Dualband Wi-Fi Antenna Element

**Figure 8:**
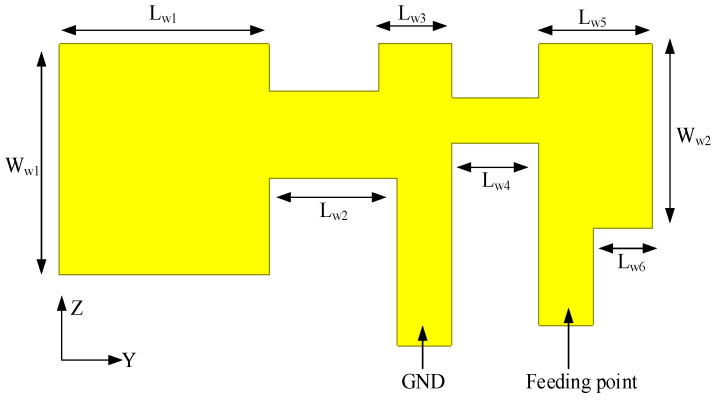
Configuration of the dualband Wi-Fi antenna element.

As illustrated in Figure 8 and summarized in Table 2, the proposed dual-band Wi-Fi antenna is derived from a modified PIFA configuration. The overall structure exhibits an inverted-F geometry, in which the outer branch serves as the feeding strip, while the inner branch functions as the shorting element. The antenna is fabricated directly from copper sheets without employing a dielectric substrate, thereby minimizing dielectric loss and improving radiation efficiency. To achieve structural miniaturization while maintaining sufficient impedance bandwidth, slotting techniques are introduced along the radiating branches. These slots effectively extend the current path within a compact physical footprint, enabling broadband dual-band operation without increasing the overall antenna volume.

**Figure 9:**
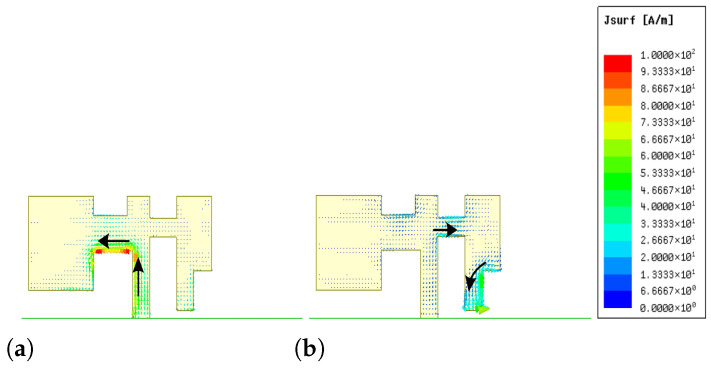
E-field of the dualband WiFi antenna element (Black arrows denote the current flow direction). (**a**) 2.45 GHz; (**b**) 5.5 GHz.

Figure 9 presents the simulated surface current distributions at representative center frequencies of the two operating bands. As shown in Figure 9a, at the lower band (2.4–2.5 GHz), strong surface currents are mainly concentrated around the left square branch. Through the intermediate rectangular connection section, the current flows toward the larger square radiator, forming an extended current loop responsible for the lower-frequency radiation. In contrast, at the upper band (5.15–5.85 GHz), as shown in Figure 9b, the current is guided through the neck-shaped rectangular section toward the blade-shaped radiating element. The dominant current concentration appears near the lower portion of the blade structure, indicating that the higher-frequency resonance is primarily supported by this localized radiating segment. The distinct current paths observed at the two frequencies confirm that the dual-band operation is realized through spatially separated resonant branches within the compact PIFA-derived structure.

#### 2.2.3. FM Antenna Element

**Figure 10:**
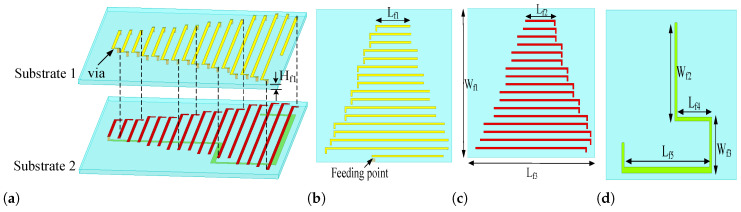
Configuration of the FM antenna element. (**a**) Perspective view; (**b**) Top side of the first layer; (**c**) Top side of the second layer; (**d**) Bottom side of the second layer.

The overall configuration and dimensional parameters of the FM antenna are illustrated in Figure 10 and summarized in Table 3. The antenna is implemented using a two-layer PCB structure. On the first substrate layer, the upper surface accommodates a set of curved radiating branches interconnected by metallized vias. On the second substrate, both the top and bottom layers are loaded with additional curved branches and vias, forming a vertically stacked current path. A gradual variation in branch length is adopted from top to bottom, where the strip length progressively increases along the vertical direction. By extending the lower branches, the effective current path is significantly elongated within a limited physical height, thereby enabling resonance within the FM band. This length-gradient configuration allows the antenna to achieve the required electrical size while maintaining compact structural dimensions. As indicated in Figure 10, the maximum horizontal length of the antenna is 47 mm, whereas the overall height is only 40 mm, demonstrating effective miniaturization for low-frequency operation. The antenna is conformally mounted at the central position of the shark-fin housing, forming the highest ridge of the integrated antenna module. All geometric parameters are labeled in the figure. The topmost branch has a length of $L_{f1}$ = 13 mm, while the bottom branch reaches $L_{f5}$ = 38 mm, with intermediate branches following a similar incremental pattern.

**Figure 11:**
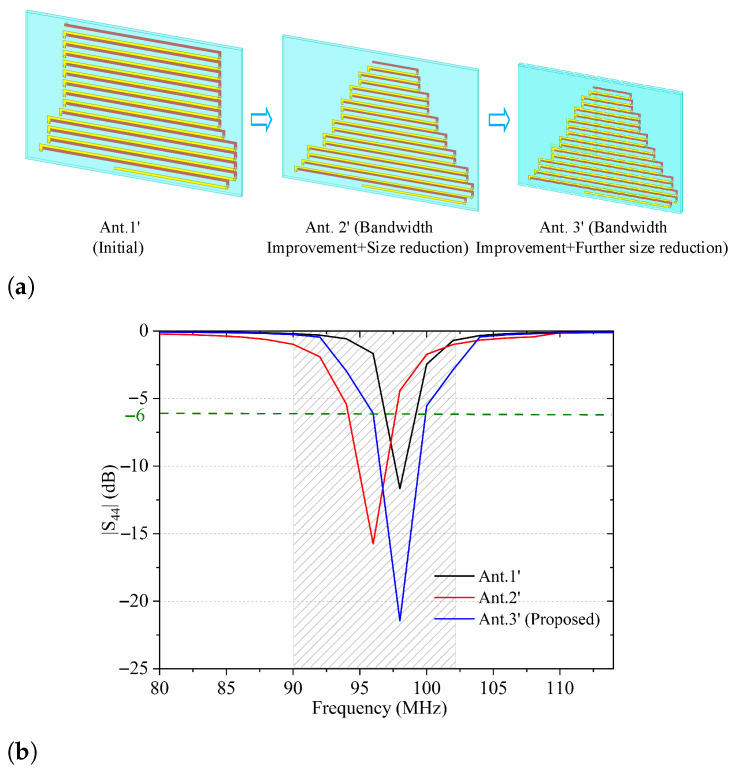
Design evolution of the proposed FM antenna element and its corresponding $\left|S_{44}\right|$ response. (**a**) element configuration at different design stages; (**b**) simulated $\left|S_{44}\right|$ magnitude.

Figure 11a depicts the design evolution of the proposed FM antenna element. In the initial stage, a uniform meandered-line configuration (Ant. 1^′^) was adopted to reduce the resonant frequency by extending the effective current path within a limited physical height. As shown by the black curve in Figure 11b, Ant. 1^′^ achieves resonance near 98 MHz. However, the impedance bandwidth is relatively narrow and fails to fully cover the FM band. The in-band matching performance is also moderate, with the minimum $|S_{44}|$ reaching only approximately −13 dB. These results indicate that conventional uniform meandered structures exhibit limited bandwidth enhancement capability at low frequencies. To improve impedance matching and further reduce the antenna footprint, a trapezoidal gradient configuration was introduced in Ant. 2^′^. Specifically, the lengths of the meandered strips increase progressively from top to bottom, forming a “narrow-top, wide-bottom” structure. This length-gradient arrangement improves the smoothness of input impedance variation with frequency and enhances impedance bandwidth. As indicated by the red curve in Figure 11b, the bandwidth is significantly expanded to 92–102 MHz, while the reduced substrate area contributes to additional miniaturization. To achieve further size reduction, Ant. 3^′^ incorporates an additional extended trace at the bottom of the second substrate layer. This added segment increases the effective electrical length without increasing the overall height. By reallocating part of the current path to the lower layer, the length of the upper trapezoidal meandered section can be shortened while maintaining the 98 MHz resonant frequency. As shown by the blue curve in Figure 11b, this configuration not only achieves improved compactness but also preserves, and even enhances, the wideband characteristic, functionally supporting the FM band.

#### 2.2.4. TETRA Antenna Element

In this work, the −6 dB (VSWR <3) criterion was intentionally adopted to evaluate the operational bandwidth, which is commonly used in compact multiband vehicular and Wi-Fi-oriented antenna designs where trade-offs between size, bandwidth, and integration complexity are required. This choice is particularly relevant for highly integrated shark-fin antenna structures, where slightly relaxed matching criteria are often employed to achieve broader functional coverage within severe spatial constraints.

**Figure 12:**
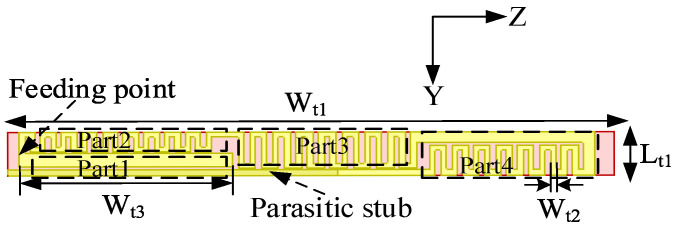
Configuration of the TETRA antenna element.

Figure 12 and Table 4 present the structural configuration and principal dimensions of the proposed TETRA antenna. The antenna is implemented using a planar printed circuit board (PCB) process and features a narrow strip-shaped geometry, with a width of $L_{t1}$ = 8 mm and a length of $W_{t1}$ = 102 mm. This compact and slender profile enables conformal integration into the rear section of the shark-fin module under strict space constraints, while exerting negligible impact on vehicular aerodynamic performance. The antenna consists of a meandered main radiator (Parts 2–4) and a parasitic straight microstrip branch (Part 1). The meandered configuration effectively extends the current path within the limited physical length, allowing the electrical length to approach a quarter wavelength and thereby achieving resonance within the low-frequency TETRA band. The parasitic branch, located near the feeding point, electromagnetically couples with the main radiator, introducing additional resonant modes that enhance impedance bandwidth and improve input matching. This combined configuration enables low-profile operation while maintaining quasi-omnidirectional radiation characteristics. Due to the electrically small nature of the FM and TETRA radiators within the confined shark-fin volume, their performance is inherently governed by the Chu–Harrington limit. The meandered-line configurations forcibly extend the current paths to reduce the resonant frequency. While this effectively decreases the electrical size, it inevitably increases the quality factor (Q-factor) and introduces additional ohmic losses, thereby imposing a strict physical trade-off among impedance bandwidth, physical footprint, and radiation efficiency.

**Figure 13:**
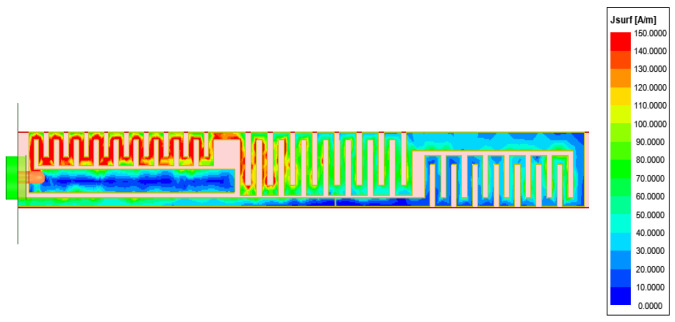
E-field of the TETRA antenna element.

The simulated surface current distribution is illustrated in Figure 13. High current density is observed near the feeding port, the initial section of the meandered line (Part 2), and the parasitic branch (Part 1), indicating that these regions dominate radiation. The current amplitude gradually decreases toward the terminal section (Part 4), exhibiting a standing-wave distribution characteristic of a quarter-wavelength monopole. This confirms that the meandered structure effectively increases the electrical length to support low-frequency resonance. Moreover, the strong current induced on the parasitic branch verifies effective electromagnetic coupling between the main radiator and the parasitic element, which excites additional resonant modes and broadens the operational bandwidth.

**Figure 14:**
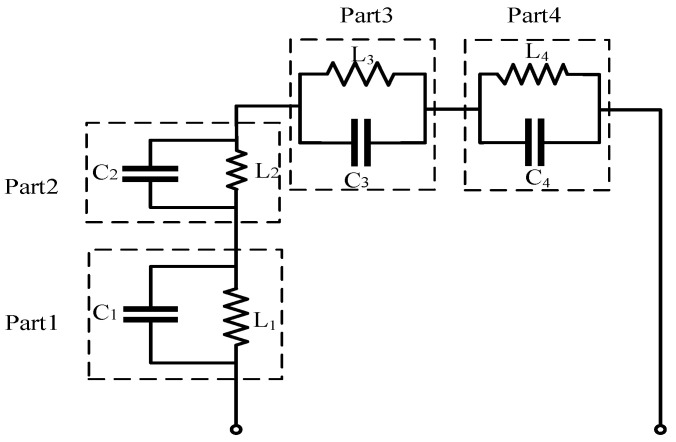
Equivalent-circuit of the TETRA antenna element.

The equivalent circuit of the TETRA antenna is shown in Figure 14. The structure is modeled as four cascaded sections (Parts 1–4), where each folded meander segment is represented by a parallel LC resonant cell. The extended current path introduces inductance, while the gaps between adjacent traces generate parasitic capacitance, jointly forming multiple resonances within the 380–470 MHz band. The parasitic rectangular branch acts as an impedance tuning element to adjust resonance depth and bandwidth, enabling low-frequency coverage within a compact footprint.

The model consists of four transmission-line sections with electrical length $\theta_{i}$ and impedance $Z_{i}$, three shunt capacitors ($C_{2}$–$C_{4}$), and one series inductor ($L_{4}$). Using the ABCD formalism [18], each transmission-line section is expressed as

(3) $$T_{Pi}=\left[\begin{array}{cc} \cos\theta_{i} & jZ_{i}\sin\theta_{i}\\ jY_{i}\sin\theta_{i} & \cos\theta_{i} \end{array}\right],$$

while the reactive elements follow standard shunt and series matrix forms. Cascading all sub-matrices yields

(4) $$T_{total}=\left[\begin{array}{cc} A & B\\ C & D \end{array}\right],$$

from which the input impedance is obtained as

(5) $$Z_{in}\left(\omega \right)=\frac{AZ_{L}+B}{CZ_{L}+D}.$$

This circuit abstraction clarifies the mechanism of resonance allocation and impedance evolution. By independently tuning reactive loading and electrical lengths, controllable resonance splitting and bandwidth enhancement can be achieved, which is particularly advantageous for compact multi-band vehicular antenna integration.

Both the FM and TETRA radiators operate as electrically small antennas (ESAs), and are therefore fundamentally constrained by the Chu limit, which imposes inherent trade-offs among electrical size, achievable bandwidth, and radiation efficiency.

## 3. Simulated and Measured Results Discussion

**Figure 15:**
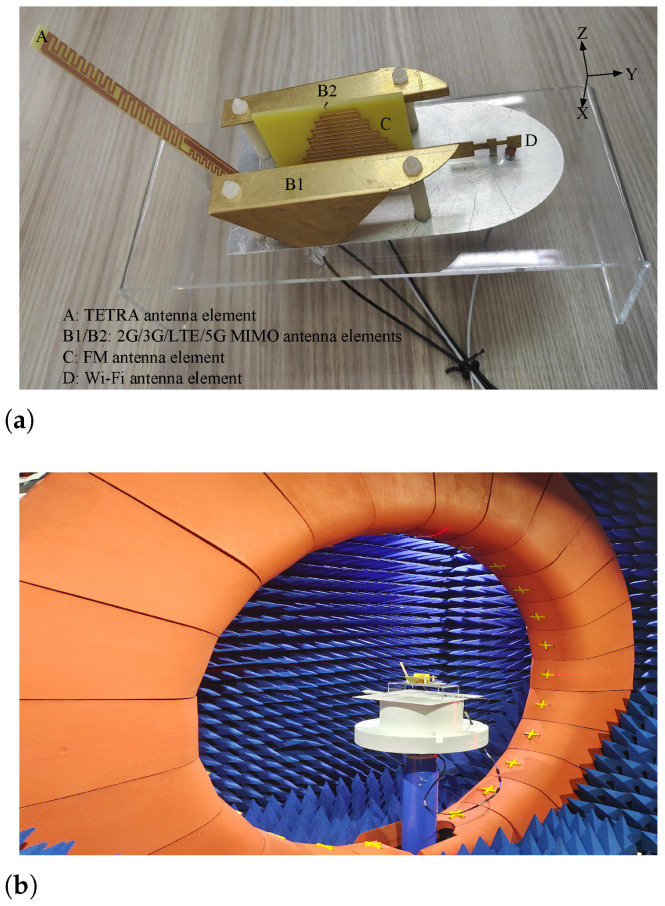
Fabricated antenna prototype and measurement setup. (**a**) Fabricated prototype; (**b**) Experimental measurement configuration.

To validate the proposed design, a prototype of the multifunctional vehicular antenna was fabricated and experimentally characterized, as shown in Figure 15a. The antenna integrates four communication modules—TETRA, 2G/3G/LTE/5G, FM, and WiFi—within a compact shark-fin enclosure. All functional elements are densely packed within a constrained volume and mechanically secured with plastic screws to ensure both structural robustness and electrical isolation. The prototype was subsequently measured in a spherical near-field anechoic chamber (40 MHz–8 GHz), manufactured by Shanghai Yimai Technology Co., Ltd. (Shanghai, China), as illustrated in Figure 15b. To emulate realistic vehicular installation conditions, a metallic ground plane was mounted beneath the antenna to represent a vehicle roof, ensuring that the measurements reflect practical on-vehicle deployment scenarios with strong engineering relevance. By incorporating an equivalent vehicle-roof ground plane within a standard anechoic measurement setup, both intrinsic antenna performance and installation-dependent radiation stability were systematically evaluated, providing comprehensive system-level validation for compact multi-service vehicular antenna integration. Furthermore, key parameter measurements were conducted to compare and correlate the simulated and experimental results.

The 3D full-wave electromagnetic simulations were performed using Ansys HFSS (High-Frequency Structure Simulator), manufactured by Ansys, Inc. (Canonsburg, PA, USA), which is based on the finite element method (FEM) in the frequency domain. HFSS was selected due to its high accuracy in modeling complex electrically small three-dimensional structures. In particular, its adaptive tetrahedral meshing strategy was employed to ensure accurate geometric discretization of the strongly curved meandered traces (used in the TETRA and FM elements) as well as the multilayer dielectric substrates densely integrated within the compact shark-fin enclosure, thereby enabling high-fidelity broadband simulation results.

All functional elements are densely packed within a constrained volume and mechanically secured using non-conductive plastic screws to minimize unintended metallic interference with the antenna’s near-field radiation. Furthermore, it should be noted that minor fabrication tolerances and manual assembly variations, such as slight misalignments of the meandered PCB elements, are the primary factors contributing to the small frequency shifts observed between the simulated and measured results in the low-frequency bands. These practical considerations ensure both structural robustness and the fidelity of the experimental validation.

**Figure 16:**
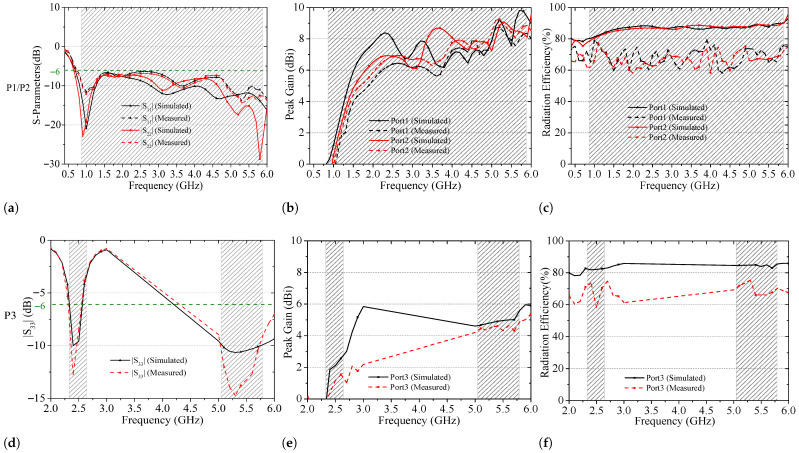

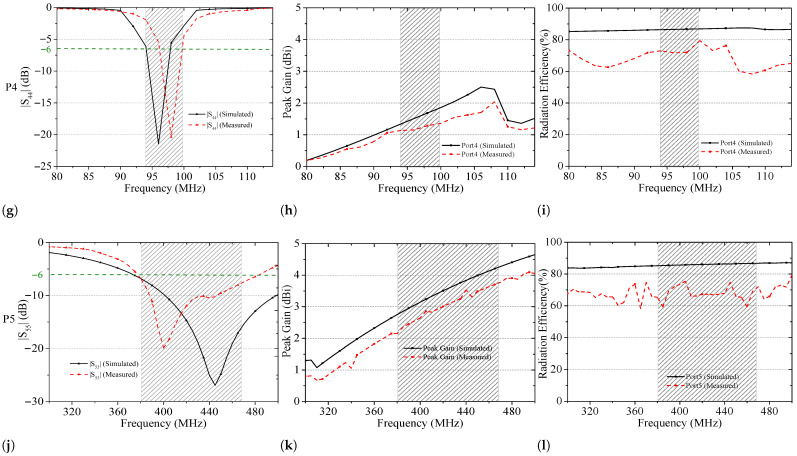
Simulated and measured bandwidths, gains, and radiation efficiency of the antenna elements. (**a**) $|S_{11}|$/$|S_{22}|$; (**b**) Peak gain; (**c**) Radiation efficiency; (**d**) $|S_{33}|$; (**e**) Peak gain; (**f**) Radiation efficiency; (**g**) $|S_{44}|$; (**h**) Peak gain; (**i**) Radiation efficiency; (**j**) $|S_{55}|$; (**k**) Peak gain; (**l**) Radiation efficiency.

Figure 16a–c present the simulated and measured electrical performance results of the 2G/3G/LTE/5G antenna element. As shown in Figure 16a, good agreement is achieved between simulation and measurement, with consistent performance at Port 1 and Port 2. The measured $|S_{11}|$ and $|S_{22}|$ remain below −6 dB from 0.68 to 6.05 GHz, corresponding to a 132.5% relative bandwidth, fully covering the required 2G/3G/LTE/5G sub-6 GHz bands. Minor deviations are attributed to measurement uncertainties, while overall performance satisfies the design targets. The measured peak gain and radiation efficiency are shown in Figure 16b,c. With the exception of frequencies below 1.2 GHz, the measured gain exceeds 5 dBi across most of the operating band. Although fabrication tolerances and dielectric losses slightly reduce efficiency compared with simulations, the measured efficiency remains above 60%, meeting vehicular application requirements.

Figure 16d–f illustrate the WiFi antenna performance. Measurements were conducted using a VNA in an anechoic chamber, with a 1 m × 1 m metallic ground plane placed beneath the antenna to emulate the vehicle roof. As shown in Figure 16d, the measured $|S_{33}|$ agrees well with simulation. The resonance depth reaches approximately −13 dB at 2.4–2.5 GHz and about −15 dB at 5.1–5.8 GHz, satisfying dual-band impedance matching requirements. The measured peak gain is about 1.5 dBi in the lower band and 5 dBi in the upper band, with radiation efficiency ranging from 55% to 70%, which fulfills practical deployment needs.

Figure 16g–i compare the FM antenna results. A small frequency shift (about 2 MHz) is observed, mainly due to fabrication tolerances and SMA parasitics. Nevertheless, the measured resonance depth reaches approximately −20 dB, and $|S_{44}|$ remains below −6 dB across the FM band. The measured peak gain ranges from 1.0 to 1.8 dBi, and the efficiency varies between 60% and 80%, which is sufficient for reception-oriented FM operation.

Finally, Figure 16j–l present the TETRA antenna performance, with unused ports terminated by 50-Ω loads. A slight downward frequency shift toward 400 MHz is observed due to fabrication and connector effects. Despite this, $|S_{55}|$ remains below −6 dB within 380–470 MHz. The measured peak gain exceeds 2 dBi (2.1–3.8 dBi), and the efficiency ranges from 60% to 75%. Overall, the measured results validate the stable multi-band performance and integration feasibility of the proposed compact V2X-oriented shark-fin antenna system.

The radiation efficiency, which remains around 70% across most operating bands, is primarily governed by a combination of dielectric and conductor losses. The use of FR-4 substrates (tanδ≈0.02) for the TETRA, FM, and Wi-Fi elements introduces non-negligible dielectric loss compared to air-dielectric structures. In addition, conductor (ohmic) loss is more pronounced in the FM and TETRA elements due to their meandered-line configurations. These miniaturized designs extend the current paths within a limited physical footprint, which increases the effective resistance and slightly reduces the overall radiation efficiency.

**Figure 17:**
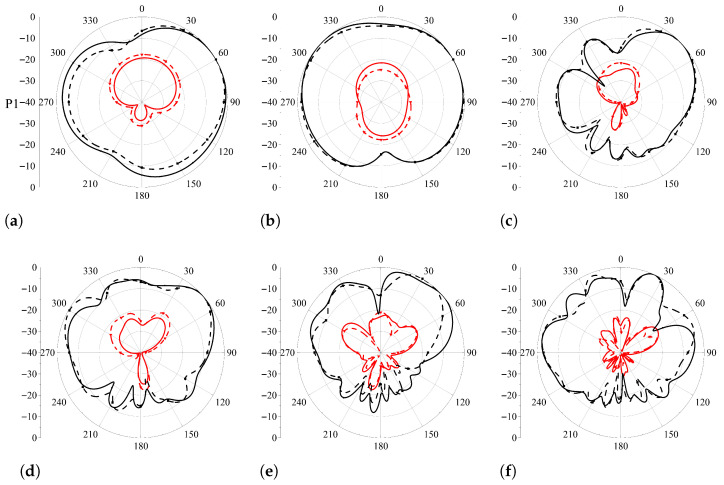

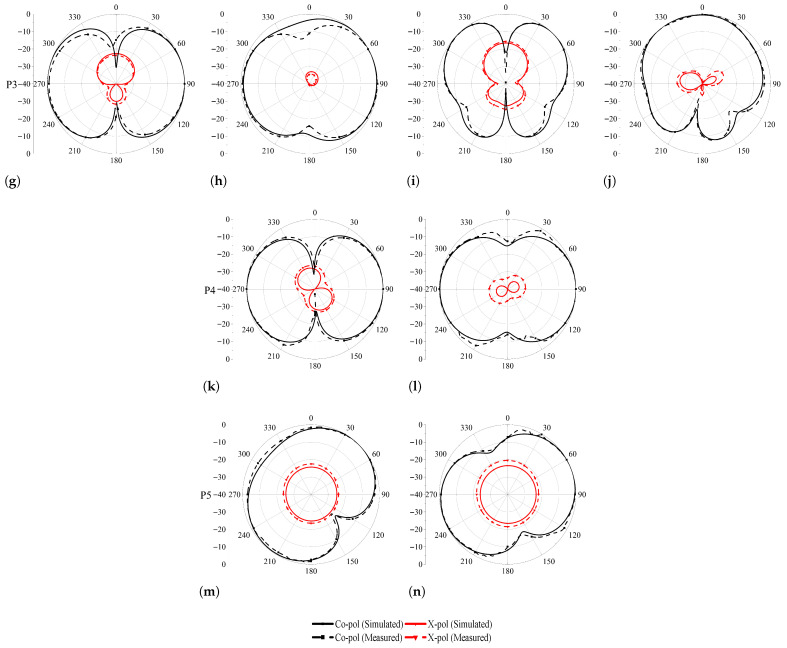
Simulated and measured radiation patterns of the proposed antenna at different frequencies. (**a**) 0.9 GHz in XOZ-plane; (**b**) 0.9 GHz in YOZ-plane; (**c**) 3.3 GHz in XOZ-plane; (**d**) 3.3 GHz in YOZ-plane; (**e**) 5.9 GHz in XOZ-plane; (**f**) 5.9 GHz in YOZ-plane; (**g**) 2.4 GHz in XOZ-plane; (**h**) 2.4 GHz in YOZ-plane; (**i**) 5.5 GHz in XOZ-plane; (**j**) 5.5 GHz in YOZ-plane; (**k**) 97 MHz in XOZ-plane; (**l**) 97 MHz in YOZ-plane; (**m**) 430 MHz in XOZ-plane; (**n**) 430 MHz in YOZ-plane.

The radiation patterns and corresponding characteristics of the proposed antenna were evaluated across all ports to assess directional performance, polarization purity, and gain stability, as shown in Figure 17 and summarized in Table 5. For the 2G/3G/LTE/5G element (Port 1), the XOZ- and YOZ-plane cross-polarization levels remain below −15 dB for all examined frequencies at 0.9 GHz (see Figure 17a,b), 3.3 GHz (see Figure 17c,d), and 5.9 GHz (see Figure 17e,f), with measured values closely matching simulations (e.g., −16.3 dB at 0.9 GHz XOZ-plane). Simultaneously, gain variations across the main lobes are limited to approximately 0.7–1.8 dB, ensuring quasi-omnidirectional coverage suitable for vehicular communications and V2X links. Port 2, being symmetrical to Port 1, exhibits comparable directional stability and polarization purity. The Wi-Fi element (Port 3) demonstrates cross-polarization levels below −15.5 dB in both XOZ- and YOZ-planes at 2.4 GHz (see Figure 17g,h) and 5.5 GHz (see Figure 17i,j), while gain fluctuation remains within 1–1.6 dB, satisfying the uniform coverage requirements of in-vehicle Wi-Fi and V2I communication scenarios. Minor discrepancies between simulated and measured patterns, e.g., 0.1–0.2 dB higher cross-polarization in measurements, are within acceptable tolerances and do not impair system performance. For the FM (Port 4) and TETRA (Port 5) elements, the radiation patterns exhibit cross-polarization levels below −19 dB at 90 MHz and 400 MHz (see Figure 17k,l for FM and Figure 17m,n for TETRA), with gain variations constrained below 0.8 dB. Such characteristics ensure robust broadcast reception and professional mobile radio connectivity even under vehicular dynamics. Overall, the measured deviations from simulations are minimal, confirming stable radiation behavior and validating the proposed design as capable of providing reliable, multi-standard coverage within a compact shark-fin form factor.

**Figure 18:**
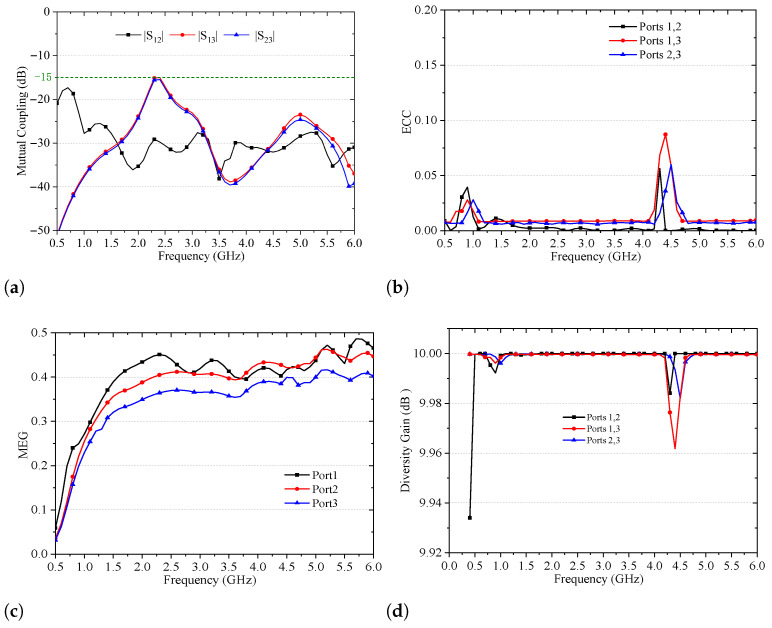
Measured mutual coupling, ECC and MEG among the ports 1, 2 and 3. (**a**) Mutual coupling; (**b**) ECC; (**c**) MEG; (**d**) Diversity gain.

Because the 2G/3G/LTE/5G sub-6 GHz MIMO elements (Ports 1 and 2) partially overlap with the dual-band Wi-Fi element (Port 3), inter-port coupling is carefully evaluated. As illustrated in Figure 18a, ∣$S_{12}$∣ remains lower than −17 dB across 0.5–6 GHz. Owing to structural symmetry, ∣$S_{13}$∣ and ∣$S_{23}$∣ almost overlap and are consistently lower than −15 dB within the operating bands. Such isolation effectively suppresses coupling-induced efficiency degradation and pattern distortion, ensuring stable multiband MIMO performance in compact vehicular platforms. The envelope correlation coefficient (ECC), extracted from the three-dimensional radiation patterns [19,20], is shown in Figure 18b. Over 0.5–6 GHz, all port pairs exhibit ECC values lower than 0.085, with nearly identical variation trends. The uniformly low correlation confirms strong channel decorrelation capability and reflects the high electromagnetic symmetry of the array configuration and feeding structure, which is essential for reliable diversity and spatial multiplexing in V2X environments. Figure 18c presents the mean effective gain (MEG). Ports 1–3 exhibit closely matched responses across the full band, and all MEG values remain below 0.5, satisfying the balanced power reception condition in multipath propagation environments [20]. The consistent MEG behavior indicates uniform power distribution, high radiation efficiency, and negligible additional loss due to mutual coupling. As illustrated in Figure 18d, the diversity gain for all port configurations remains consistently high across the entire operating band, approaching the ideal value of 10 dB. This indicates near-optimal mitigation of multipath fading and effective utilization of spatial diversity. Notably, the variation is confined within ±0.5 dB of the theoretical maximum over a wide frequency range from sub-GHz to C-band, demonstrating stable and broadband performance. Such results are seldom achieved in practical multi-antenna systems, particularly without employing complex decoupling or matching networks. These findings confirm the excellent spatial correlation characteristics of the proposed design, enabling reliable and high-throughput MIMO operation in rich-scattering environments. From an engineering standpoint, the sustained near-ideal diversity gain translates into improved link reliability, reduced fading margins, and enhanced spectral efficiency.

Overall, the proposed architecture achieves sufficient isolation, low ECC, balanced effective gain, and high diversity gain, validating its suitability for integrated V2X-oriented communication systems.

To place the proposed shark-fin antenna in context, a comparative evaluation with representative state-of-the-art vehicular antennas is summarized in Table 6. The comparison considers antenna architecture, operational bandwidth, physical dimensions, MIMO capability, radiation efficiency, number of supported bands, and key radiation performance metrics.

Kwon et al. [5] reported a compact shark-fin antenna integrating MIMO-LTE, GPS, Wi-Fi, and WAVE functionalities. The configuration, consisting of two PIFAs and modified planar monopoles, occupies a volume of 59.5×44.3×21 mm^3^ ($0.39\lambda_{0}\times 0.29\lambda_{0}\times 0.14\lambda_{0}$), achieving an isolation level of approximately 10 dB and an ECC below 0.2. The antenna supports four distinct services. However, the radiation efficiency is only 30.7%, and the operating bands (0.85–0.96 GHz and 1.71–2.69 GHz) do not fully cover the sub-6 GHz 5G spectrum, indicating limited scalability. Liu et al. [21] proposed a four-element shark-fin module integrating LTE main/diversity, FM, and GPS antennas. By adopting an aggressive miniaturization strategy, a compact profile of 55×25×2 mm^3^ was achieved, with isolation exceeding 10 dB, ECC below 0.15, and radiation efficiency of 60%. Although the design covers three frequency bands, the upper operating frequency is limited to 2.69 GHz, excluding higher 5G NR FR1 bands. In addition, the FM radiator exhibits a gain of approximately −25 dBi, limiting its effectiveness in V2X scenarios. Khalifa et al. [22] introduced a single-element wideband monopole covering 0.617–5 GHz, corresponding to a 156% fractional bandwidth. The antenna achieves a radiation efficiency of 83% and incorporates an L-shaped stub for GNSS interference suppression. Although the average realized gain remains above −1 dBi, the single-port configuration does not support MIMO diversity, and the 60 mm profile height limits integration in low-profile vehicular platforms. Zhou et al. [23] developed a 2×2 MIMO antenna system covering 0.617–0.96 GHz and 1.71–6 GHz using multi-branch monopoles and parasitic elements. The reported isolation ranges from 6 to 10 dB with ECC values between 0.2 and 0.4. Although full sub-6 GHz coverage is achieved, radiation efficiency is not reported, and the relatively low isolation in the lower band together with impedance mismatch at higher frequencies may limit MIMO throughput. Moreover, the overall size of 48.5×30×49.5 mm^3^ remains relatively large for dense integration in shark-fin platforms.

By contrast, the proposed design integrates five radiating elements within a compact envelope of 90×15×30 mm^3^, enabling simultaneous support for FM, TETRA, wideband cellular (2G–5G), and dual-band Wi-Fi services. It achieves an average radiation efficiency of approximately 70%, true 2×2 MIMO operation with ECC below 0.1, and inter-port isolation exceeding 15 dB. In addition, the antenna exhibits a peak realized gain of up to 7 dBi while maintaining a low-profile structure.

The main design trade-offs arise from the increased electrical length required for low-frequency radiators and high-frequency coexistence, as well as a minor impedance mismatch in adjacent non-target bands, resulting in a realized gain reduction of approximately 0.8 dB. Overall, the proposed antenna provides a compact, high-isolation, multiband vehicular platform supporting five wireless services, significantly simplifying antenna integration in modern connected vehicle systems.

## 4. Link Budget and Margin Verification

To evaluate the system-level feasibility of the proposed multiband shark-fin antenna, a reference link budget analysis is performed. The received power is expressed as [25,26]

(6) $$P_{r}=P_{t}+G_{t}+G_{r}-L_{p}-L_{o}+G_{\mathrm{LNA}},$$

where $P_{t}$ denotes the transmit power (dBm), $G_{t}$ and $G_{r}$ represent the realized gains (dBi) of the transmitting and receiving antennas, respectively, inherently accounting for the mismatch loss factor $\left(1-|\Gamma {|}^{2}\right)$, $L_{p}$ denotes the propagation loss (dB), $L_{o}$ represents additional losses including feeder attenuation, vehicular blockage, and polarization mismatch, and $G_{\mathrm{LNA}}$ is the gain of the low-noise amplifier (LNA), included when applicable.

The free-space path loss (FSPL) is calculated as

(7) $$L_{p}=32.44+20\log_{10}\left(f_{\mathrm{MHz}}\right)+20\log_{10}\left(d_{\mathrm{km}}\right),$$

which is adopted here as a reference model.

### 4.1. Cellular Bands (1 km)

For a macro-cell scenario with $P_{t}=46$ dBm, a propagation distance of 1 km is selected to represent a typical macro-cell edge coverage condition for cellular Vehicle-to-Network (V2N) links. With base-station antenna gains of 15 dBi at 900 MHz and 17 dBi at 1800/3500 MHz, the corresponding received powers are computed using (6)–(7) as follows:

- 900 MHz: $L_{p}=91.52$ dB, $P_{r}=-31.52$ dBm;
- 1800 MHz: $L_{p}=97.55$ dB, $P_{r}=-31.55$ dBm;
- 3500 MHz: $L_{p}=103.32$ dB, $P_{r}=-38.32$ dBm.

Typical LTE/NR receiver sensitivities range from approximately −95 to −100 dBm depending on bandwidth and modulation order [27]. Therefore, the reference link margins exceed 55 dB. Considering additional vehicular shadowing and fast fading described in standardized channel models [28], sufficient system headroom is retained.

### 4.2. WiFi Bands (50 m)

For a vehicular hotspot scenario with $P_{t}=23$ dBm, a propagation distance of 50 m is selected to represent high-throughput in-vehicle connectivity and short-range Vehicle-to-Infrastructure (V2I) hotspot environments:

- 2.4 GHz: $L_{p}=74.02$ dB, $P_{r}=-49.92$ dBm;
- 5.5 GHz: $L_{p}=81.23$ dB, $P_{r}=-52.43$ dBm.

IEEE 802.11 receiver sensitivities typically vary between −82 and −90 dBm depending on the modulation and coding scheme (MCS) [29]. The calculated link margins therefore remain above 30 dB.

### 4.3. TETRA (400 MHz, 2 km)

With $P_{t}=40$ dBm and $G_{t}=G_{r}=3$ dBi, the received power is $P_{r}=-44.50$ dBm. Compared with a typical TETRA receiver sensitivity of approximately −112 dBm specified in ETSI standards [30], the resulting link margin exceeds 65 dB.

### 4.4. FM Broadcast (100 MHz, 30 km)

For $P_{t}=70$ dBm and $G_{t}=3$ dBi, the path loss is $L_{p}=101.98$ dB. With a passive antenna gain of −15 dBi and a 20 dB LNA, the received power is $P_{r}=-23.98$ dBm, providing functional broadcast reception.

### 4.5. Discussion

Under the FSPL reference model in (7), all evaluated services exhibit substantial link margins ranging from 30 dB to over 65 dB. Although practical vehicular channels introduce additional shadowing and multipath fading [28], the calculated results confirm that the proposed compact multiband antenna provides sufficient gain and radiation performance to sustain integrated V2X-oriented communication links with robust system-level tolerance.

Although their compact footprints lead to relatively low passive realized gains (1.1–1.4 dBi for the FM element and 2.1–3.8 dBi for the TETRA element), these antennas are primarily designed for reception. In practical vehicular applications, such gain limitations are effectively mitigated through the integration of active low-noise amplifiers (LNAs). Consequently, the optimized meandered configurations achieve a well-balanced trade-off within the ESA regime, while ensuring reliable and robust signal reception.

Specifically, a standard fading margin has been incorporated based on the 3GPP TR 38.901 channel model [28]. In urban microcell (UMi) non-line-of-sight (NLOS) scenarios, shadow fading is typically modeled as a log-normal distribution with a standard deviation of approximately 7.82 dB. By introducing a representative engineering fading margin in the range of 10–15 dB, it is demonstrated that the calculated baseline link margins (exceeding 30 dB for Wi-Fi and 65 dB for TETRA) remain sufficiently large. These results indicate that adequate power headroom is preserved, thereby ensuring robust and reliable communication performance even under severe multipath propagation and shadowing conditions.

Notably, the observed 40–50 MHz downward frequency shift is primarily attributed to measurement-related environmental effects. Specifically, the finite metallic roof plate and common-mode currents on the VNA coaxial cable introduce additional loading to the UHF monopole, which lowers the resonant frequency compared to the ideal infinite ground plane assumed in the HFSS simulations.

Regarding the impedance matching criterion, although −10 dB is the conventional standard, the TETRA element operates as an electrically small antenna (ESA) within a highly constrained volume. Under such conditions, achieving wideband −10 dB matching is fundamentally limited by physical bounds. Therefore, a −6 dB (VSWR <3) criterion is adopted as a practical engineering compromise for compact multiband integration.

Under this criterion, the antenna effectively covers the intended TETRA band. Moreover, the system-level evaluation is based on the measured realized gain, which inherently accounts for mismatch losses. The link budget analysis demonstrates a robust margin of up to 65 dB, while all supported services (FM, TETRA, cellular, and Wi-Fi) maintain margins ranging from 30 dB to over 65 dB. These results confirm that the adopted matching criterion is sufficient to meet and exceed the performance requirements of practical V2X communication scenarios.

Furthermore, the proposed architecture offers significant scalability for future vehicular communication systems. The strategic layout of the sub-6 GHz elements preserves unutilized physical space at the top-front and lateral edges of the enclosure. This provides valuable, integration-ready headroom for the future conformal integration of millimeter-wave (mmWave) phased arrays, facilitating the smooth evolution toward 6G V2X applications.

## 5. Conclusions

A compact multiband shark-fin antenna for integrated V2X systems has been presented and experimentally verified. The five-element architecture, confined within a 90 × 15 × 30 mm^3^ enclosure, simultaneously supports FM, TETRA, wideband 2G–5G cellular (0.68–6.05 GHz), and dual-band Wi-Fi services. Measured gains are in the range of 1.1–1.4 dBi for the FM band and 2.1–3.8 dBi for the TETRA band, while the cellular band achieves a peak gain of up to 5 dBi, and the Wi-Fi band exhibits a gain varying from −0.4 to 4.8 dBi.

Both simulations and measurements confirm stable quasi-omnidirectional radiation, cross-polarization levels below −13 dB, and inter-element isolation better than −15 dB across all bands. By integrating miniaturized low-frequency elements, mirrored wideband PIFAs, and a dual-branch IFA within a unified platform, the design achieves balanced compactness, broadband coverage, and multi-radio coexistence. The proposed antenna therefore provides an integration-ready solution for next-generation sub-6 GHz V2X applications.

## Figures and Tables

**Table 1:** Principal dimensions of the 2G/3G/LTE/5G antenna element.

Parameter	$L_{g1}$	$L_{g2}$	$L_{g3}$	$L_{g4}$	$H_{g1}$	$W_{g1}$
Value (mm)	90	4	15	65	30	15

**Table 2:** Dimensions of the dual-band Wi-Fi antenna element.

Parameter	$L_{w1}$	$L_{w2}$	$L_{w3}$	$L_{w4}$	$L_{w5}$	$L_{w6}$	$W_{w1}$	$W_{w2}$
Value (mm)	9.6	5.8	3.3	4	5	2.7	10	8

**Table 3:** Dimensions of the FM antenna element.

Parameter	$L_{f1}$	$L_{f2}$	$L_{f3}$	$L_{f4}$	$L_{f5}$	$W_{f1}$	$W_{f2}$	$W_{f3}$	$H_{f1}$
Value (mm)	13	12	47	14.6	38	40	26	14.5	1

**Table 4:** Principal dimensions of the TETRA antenna element.

Parameter	$L_{t1}$	$W_{t1}$	$W_{t2}$	$W_{t3}$
Value (mm)	8	102	0.95	37

**Table 5:** Radiation performance of different antenna ports.

Port 1 (2G/3G/LTE/5G antenna element)						
Freq (GHz)	XOZ-plane X-pol (dB)		YOZ-plane X-pol (dB)		Gain variation (dB)	
	Sim.	Meas.	Sim.	Meas.	Sim.	Meas.
0.9	−17.5	−16.3	−18.5	−17.6	0.6	0.7
3.3	−16.9	−15.5	−17.9	−17.5	1.0	1.2
5.9	−15.5	−14.5	−15.1	−13.8	1.7	1.8
**Port 3 (Wi-Fi antenna element)**						
Freq (GHz)	XOZ-plane X-pol (dB)		YOZ-plane X-pol (dB)		Gain variation (dB)	
	Sim.	Meas.	Sim.	Meas.	Sim.	Meas.
2.4	−18.5	−17.9	−17.9	−16.5	0.8	1.0
5.5	−16.2	−15.5	−16.5	−15.8	1.5	1.6
**Port 4 (FM antenna element)**						
Freq (MHz)	XOZ-plane X-pol (dB)		YOZ-plane X-pol (dB)		Gain variation (dB)	
	Sim.	Meas.	Sim.	Meas.	Sim.	Meas.
90	−20.1	−19.5	−22.5	−20.4	0.3	0.5
**Port 5 (TETRA antenna element)**						
Freq (MHz)	XOZ-plane X-pol (dB)		YOZ-plane X-pol (dB)		Gain variation (dB)	
	Sim.	Meas.	Sim.	Meas.	Sim.	Meas.
400	−20.3	−18.9	−21.5	−19.8	0.5	0.8

**Table 6:** Comparison of the proposed antenna with reported shark-fin antennas.

Ref.	Elem.	BW (%)	FR	Size (mm^3^)	Gain (dBi)	Iso. (dB)	ECC	η (%)	Bands
[5]	3	12.2/42.6	2.43	59.5×44.3×21	−1–5.1	10	0.2	30.7	4
[21]	4	22/31/42.6	22.45	55×25×2	−0.7–5	10	0.15	60	3
[22]	1	156	8.10	38.5×14.9×60	−1.5–3	N.A.	N.A.	83	1
[23]	4	43.5/111	9.72	48.5×30×49.5	N.A.	6/10	0.4/0.2	N.A.	2
[1]	1	43.5/44.9/41.0	8.1	58×30×1	2.7–5	N.A.	N.A.	80	1
[24]	1	40	1.57	60×60×8.5	7.7–8.7	N.A.	N.A.	90	1
**This work**	5	22/22.5/132.5	34.17	90×15×30	−1–7	15	0.1	70	5
Ref.	Remarks								
[5]	Compact multiband antenna; high isolation; MIMO system								
[21]	Multiband antenna; low ECC; MIMO system								
[22]	Compact wideband antenna; GNSS band rejection								
[23]	Multiband antenna; full 5G coverage; MIMO system								
[1]	Compact wideband antenna; consistent radiation pattern; CMA based								
[24]	UWB Yagi-Uda antenna; stable flat gain; vehicular 5G system								
**This work**	Multi-band; low ECC; compact size; MIMO system								

BW: bandwidth; FR: frequency ratio ($F_{uc}/F_{lc}$); Iso.: isolation; Elem.: number of elements; η: radiation efficiency. $F_{uc}$ and $F_{lc}$ denote upper and lower center frequencies, respectively. N.A.: not available.

## Data Availability

Data are contained within the article. Further inquiries can be directed to the corresponding authors.
